# Comparison of the Effects of Sugammadex Recommended Dose (2 mg/kg) and Fixed Dose of 200 mg on the Reversal of Moderate Neuromuscular Block and Recovery Profile in Adult Patients

**DOI:** 10.3390/medicina61010151

**Published:** 2025-01-17

**Authors:** Ji-Yoon Jung, Sung-Ae Cho, Woojin Kwon, Hongwook Kim, Tae-Yun Sung

**Affiliations:** 1 Department of Anaesthesiology and Pain Medicine, Konyang University Hospital, Konyang University Myunggok Medical Research Institute, Konyang University College of Medicine, Daejeon 35365, Republic of Korea; jiyooning1030@gmail.com (J.-Y.J.); 200685@kyuh.ac.kr (S.-A.C.); 200723@kyuh.ac.kr (W.K.); 2 Department of Urology, Konyang University Hospital, Konyang University College of Medicine, Daejeon 35365, Republic of Korea; urokim@kyuh.ac.kr

**Keywords:** neuromuscular blocking agents, sugammadex, neuromuscular monitoring

## Abstract

*Background and Objectives*: Neuromuscular blocking agents are essential to ensure optimal surgical conditions during general anesthesia. Sugammadex, a selective binding agent, is widely used to reverse neuromuscular blockade. While weight-based dosing (2 mg/kg for moderate blockade) is recommended, many clinicians administer a fixed dose of 200 mg in clinical practice, potentially leading to overdosing. This study aimed to compare the efficacy and recovery profiles of weight-based and fixed-dose sugammadex in patients undergoing general anesthesia. *Materials and Methods*: In this randomized controlled trial, 20 patients were divided into two groups: the recommended dose group (R group, 2 mg/kg) and the fixed dose group (F group, 200 mg). Primary outcomes included time to achieve a normalized train-of-four (TOF) ratio of 0.9. Secondary outcomes included recovery time, time to spontaneous respiration, response to verbal commands and extubation, and adverse events. *Results*: The median time to achieve a normalized TOF ratio of 0.9 was 2.3 min in the R group and 2.0 min in the F group (*p* = 0.529). Secondary outcomes, including recovery time and time to extubation showed no significant differences. Adverse events were minimal and comparable between groups. *Conclusions*: The fixed-dose sugammadex (200 mg) demonstrated similar efficacy and safety to weight-based dosing (2 mg/kg) in reversing moderate neuromuscular blockade. These findings do not allow abandoning the recommendation of adjustment for body weight, particularly in patients with low body weight or comorbidities.

## 1. Introduction

During surgery under general anesthesia, neuromuscular blocking agents are commonly administered to facilitate atraumatic intubation, ensure adequate surgical fields, and enhance the ease of the surgical procedure [1,2]. Traditionally, anticholinesterases have been used to reverse nondepolarizing neuromuscular blocking agents. However, since the introduction of sugammadex, a selective binding agent, in 2015 [3,4], its use has been recommended for the reversal of deep, moderate, and shallow depths of neuromuscular blockade according to established guidelines [5,6].

The recommended dose of sugammadex is 4 mg/kg for deep blockade and 2 mg/kg for moderate or shallow blockade. Despite these recommendations, in clinical practice, anesthesiologists often administer a fixed dose of 200 mg (1 vial) of sugammadex, regardless of the patient’s body weight or the depth of the neuromuscular blockade, as the most common “standard” dose [7,8]. This practice stems from its convenience and perceived adequacy for a broad range of patients, making it a common real-world alternative to weight-based dosing. Due to this practice, sugammadex was frequently (57%) overdosed in patients in clinical situations [8].

Previous studies have well-documented the risks associated with underdosing sugammadex, such as incomplete neuromuscular blockade reversal and recurarization [9,10]. Conversely, the impact of fixed-dose sugammadex, particularly the potential for overdosing, on the efficacy of neuromuscular blockade reversal and recovery profiles from general anesthesia remains poorly understood, with only limited reports available [11,12].

To address this gap, this study was designed to compare the effects of the recommended weight-based dose of sugammadex (2 mg/kg) versus a fixed dose of 200 mg on the reversal of moderate neuromuscular blockade and recovery profiles in patients undergoing general anesthesia. Through this randomized controlled trial, we aimed to propose evidence-based strategies to optimize patient safety and efficacy in neuromuscular blockade reversal.

## 2. Materials and Methods

This prospective randomized controlled study was approved by the Institutional Review Board (KYUH 2023-08-002) of our hospital. Written informed consent was obtained from all the participants. This protocol was registered at the Korean Clinical Research Information Service (https://cris.nih.go.kr/, KCT0008773) before participant enrolment.

We enrolled patients aged 19–65 years, body weight less than 90 kg, American Society of Anesthesiologists (ASA) physical status of I–II who underwent elective surgery under general anesthesia between November 2023 and February 2024 at a single university hospital. The inclusion criterion of body weight less than 90 kg was chosen to ensure that the fixed dose of 200 mg would not lead to significant underdosing relative to the recommended weight-based dose of 2 mg/kg. The exclusion criteria were any contraindication to sugammadex; a body mass index less than 18.5 kg/m^2^ or greater than 30 kg/m^2^; moderate or severe liver, kidney, heart, or respiratory disease; neuromuscular disorders; and cognitive impairment.

Patients were randomly assigned to either the sugammadex of recommended dose group (R group) or the fixed dose group (F group) in a 1:1 ratio using online randomization software (Researcher Randomizer; www.randomizer.org). The patients were assigned by an assistant unrelated to this study and the patient’s group allocation was concealed in a sealed opaque envelope, which was revealed 10 min before the anticipated end of the surgery by the attending anesthesiologist who administered sugammadex.

All patients fasted for at least 8 h and entered the operating room without premedication. Standard monitoring, including pulse oximetry, non-invasive blood pressure, electrocardiography, bispectral index (BIS; BIS VISTA™ monitor; Aspect Medical Systems, Norwood, MA, USA), and Bair-Hugger^TM^ temperature monitoring system were used. Neuromuscular monitoring was carried out according to international guidelines [13]. For neuromuscular monitoring, a TOF-Watch-SX^®^ acceleromyograph (Organon Ltd., Dublin, Ireland) was used on the adductor pollicis muscle of the arm opposite to the one used for blood pressure measurement.

Anesthesia was induced with propofol (2–2.5 mg/kg) and fentanyl (1–2 μg/kg). After loss of consciousness, TOF-Watch-SX^®^ was calibrated, and train-of-four (TOF) stimulation (supramaximal stimuli, 0.2 ms; frequency, 2 Hz) was continued every 15 s. After signal stabilization (i.e., less than 5% variability in the amplitude of the response to nerve stimulation for at least 2 min), baseline TOF ratio was recorded. Rocuronium (0.6 mg/kg) was administered to facilitate endotracheal intubation. Anesthesia was maintained with an O_2_/50% nitrous oxide mixture and the end-tidal concentration of desflurane was adjusted to maintain a target BIS of 40–60. During anesthesia, the end-tidal carbon dioxide concentration was controlled in the range of 35–45 mmHg through mechanical ventilation, and the core temperature was maintained above 35 °C using forced-air warming. Intraoperative rocuronium was injected at a dose of 0.1–0.2 mg/kg whenever clinically required or when the second twitch (T2) of TOF response reappeared to achieve a moderate depth of neuromuscular block (i.e., TOF count 1–3) at the end of the surgery. During surgery, TOF stimulation was performed every 5 min, but after sugammadex administration, it was performed every 15 s.

After the completion of surgery, the inhalation anesthetic was discontinued, and the patient was ventilated with 100% O_2_ at 6 L/min. Following this, according to the assigned group: the R group received 2 mg/kg of sugammadex, the recommended dose for reversal of moderate neuromuscular blockade, while group F received a fixed dose of 200 mg. The fixed dose of 200 mg was chosen as it is widely used in clinical practice as a convenient “standard” dose for moderate neuromuscular blockade reversal [7,8]. In both groups, TOF count, BIS value, age-adjusted minimal alveolar concentration (MAC), and core temperature were recorded at the time of sugammadex administration. The time taken from injection of sugammadex to normalized TOF ratio of 0.9, spontaneous respiration, response to verbal commands, and extubation were measured. The normalized TOF ratio is calculated as displayed TOF ratio at recovery/baseline TOF ratio. Additionally, bradycardia (i.e., heart rate < 50 beats/min), airway obstruction (i.e., case requiring continuous [>30 s] positive pressure ventilation with or without an airway device), and cough (0 = no cough; 1 = mild, single cough; 2 = moderate, more than 1 lasting for <5 s; 3 = severe, sustained cough for 5 s) were evaluated from the time of sugammadex administration until 5 min after extubation.

The primary outcome of this study was the time taken from injection of sugammadex to a normalized TOF ratio of 0.9, which is the time at which the first of three consecutive TOF was recorded. Secondary outcomes were recovery time (i.e., time from the end of surgery to extubation), time from sugammadex injection to spontaneous respiration, response to verbal command, and extubation, incidence of airway obstruction, and severity of cough.

### Statistical Analyses

In previous studies [14,15], the mean time to reach a TOF ratio of 0.9 after the administration of sugammadex was 2 min (standard deviation of 0.7 min) when 2 mg/kg of sugammadex was administered in moderate neuromuscular block. Based on these data, the sample size was calculated based on the assumption that fixed dose of sugammadex (200mg) would shorten the time by 50%. With an effect size *d* of 1.429, a α-value of 0.05 (two-tailed), power of 0.8, and an allocation ratio of 1:1, 9 patients per group were required. Considering a potential dropout, 10 patients per group were enrolled in this study. To account for potential variability in individual responses to sugammadex, the inclusion and exclusion criteria were designed to minimize confounding factors and variability, such as excluding patients with a body mass index (BMI) outside the range of 18.5–30 kg/m^2^, significant comorbidities, or neuromuscular disorders.

We used the SPSS Statistics software (ver. 27.0 for IBM Corp., Armonk, NY, USA) for the statistical analyses. Continuous variables were compared using Student’s *t*-test or Mann–Whitney U test after assessing the data distribution using the Kolmogorov–Smirnov test. Categorical variables were compared using Fisher’s exact test or χ^2^ test for trends (linear-by-linear association), as appropriate. A *p*-value of <0.05 was considered statistically significant.

## 3. Results

**Figure 1:**
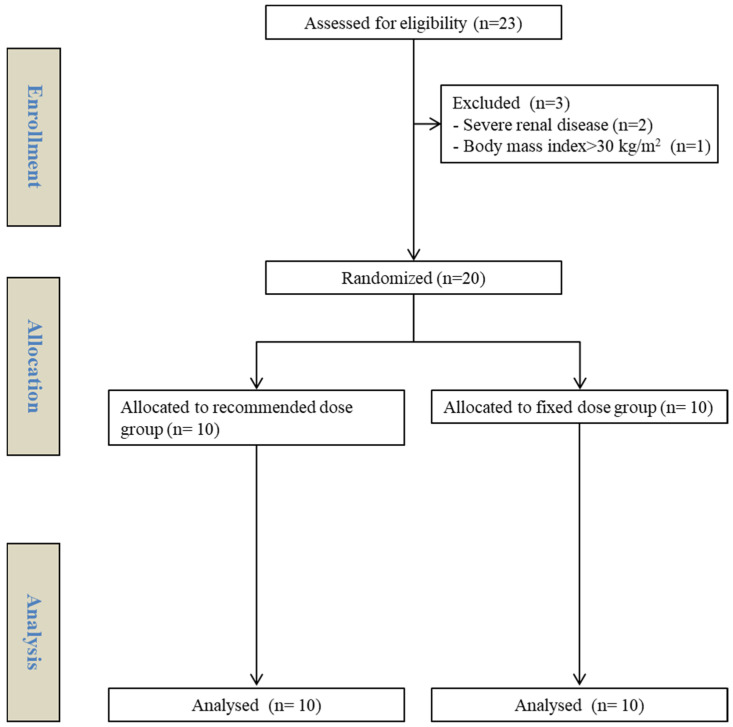
CONSORT participant flow chart.

In total, 23 patients were assessed for eligibility; 3 patients were excluded because of severe renal disease (n = 2) and BMI > 30 kg/m^2^ (n = 1). Thus, 20 patients were randomly assigned to either the sugammadex of recommended dose group (R group) or the fixed dose group (F group). All patients received the assigned intervention and were included in the final analysis (Figure 1). The demographics and baseline characteristics shown in Table 1 were comparable between the two groups.

The intraoperative data and results of primary and secondary outcomes are shown in Table 2. The median time taken from the injection of sugammadex to achieve a normalized TOF ratio of 0.9 was 2.3 min [IQR 1.8–2.9] in the R group and 2.0 min [IQR 1.6–2.6] in the F group, showing no significant difference (*p* = 0.529).

Additionally, recovery time (7.0 ± 1.6 min in the R group vs. 6.1 ± 2.1 min in the F group, *p* = 0.296), the time from sugammadex injection to spontaneous respiration (4.8 ± 2.0 min in the R group vs. 4.0 ± 2.3 min in the F group, *p* = 0.420), response to verbal commands (5.8 ± 1.7 min in the R group vs. 5.3 ± 1.9 min in the F group, *p* = 0.521), and extubation (5.9 ± 1.6 min in the R group vs. 5.5 ± 2.0 min in the F group, *p* = 0.629) showed no significant differences between the two groups.

Furthermore, no cases of airway obstruction or bradycardia occurred in either group and, the grades of cough also showed no significant differences, indicating no differences in the occurrence of adverse events between the two groups.

## 4. Discussion

This study aimed to compare the effects of a fixed sugammadex dose of 200 mg and a weight-based recommended dose of 2 mg/kg on the reversal of moderate neuromuscular blockade and the recovery profile in patients undergoing general anesthesia. The time to reach a normalized TOF ratio of 0.9 showed no significant difference between the administration of 2 mg/kg of sugammadex and the standard fixed dose of 200 mg for moderate blockade. Additionally, the secondary outcomes, including recovery time, the time from sugammadex injection to spontaneous respiration, response to verbal commands, extubation, the incidence of airway obstruction, and the severity of cough, showed no significant differences between the two groups.

The guidelines on monitoring and antagonism of neuromuscular blockade, published in 2023 in the United States and Europe, provide strong recommendations based on high-level evidence for the use of sugammadex over neostigmine in reversing deep, moderate, and shallow depths of neuromuscular blockade induced by aminosteroidal neuromuscular blocking agents such as rocuronium and vecuronium [5,6].

Although these guidelines do not provide precise recommendations regarding sugammadex dosing [5,6], previous reports indicate that the dose of sugammadex required for sufficient reversal of neuromuscular blockade to allow extubation and patient recovery varies depending on the depth of the neuromuscular blockade [6]. For deep neuromuscular blockade, characterized by a Post-Tetanic Count (PTC) ≥ 1 and a Train-of-Four (TOF) count of 0, a dose of 4 mg/kg is recommended. For moderate neuromuscular blockade, with a TOF count of 1–3, a dose of 2 mg/kg is advised [5,13]. When the appropriate dose is administered, recovery of the Train-of-Four (TOF) ratio to 0.9 or higher can be expected on average within 2 to 3 min [10,13].

Based on these principles and guidelines, it is evident that when neuromuscular blocking agents are used during surgery, intraoperative neuromuscular monitoring is essential to assess the depth of blockade. Additionally, during reversal, the appropriate dose of a reversal agent should be administered based on the neuromuscular monitoring to ensure optimal recovery.

However, according to the results of a multicenter survey, the dosing of sugammadex is often based on a fixed dose rather than on TOF monitoring results or body weight. Reversal agents were administered more frequently based on clinical signs rather than objective monitoring results, and a significant percentage of cases reported insufficient recovery of the TOF ratio at the time of extubation or residual neuromuscular blockade [7]. Another survey revealed that in clinical practice, a significant number of anesthesiologists most commonly administer 200 mg of sugammadex as the “standard” dose which may constitute a mild overdose according to the official prescription information [8].

The reasons behind such clinical practice can be speculated as follows:

Although various tools and devices for monitoring the depth of neuromuscular blockade have been introduced [13], neuromuscular monitoring is still not commonly performed in clinical settings [7]. In such cases, to prevent incomplete recovery or residual neuromuscular blockade [16], a dose of 200 mg, equivalent to one vial, is often administered under the assumption of at least a moderate blockade, even if sufficient time has elapsed since the administration of the neuromuscular blocking agent.

Additionally, since sugammadex is dose-dependent in effectively reversing neuromuscular blockade, anesthesiologists may prefer to administer the entire vial (200 mg) rather than titrating the dose according to the depth of the blockade, following established practices.

When monitoring the depth of neuromuscular blockade, neuromuscular blocking agents are typically administered to maintain moderate blockade during surgery unless deep blockade is specifically required to improve surgical conditions. As a result, at the end of surgery, the depth of neuromuscular blockade is often in a state of moderate or shallow blockade. Based on guidelines recommending 2 mg/kg of sugammadex for moderate blockade [17,18], patients weighing between 50 kg and 90 kg may receive an overdose ranging from as little as 20 mg to as much as 100 mg compared to the recommended dose.

While previous studies have reported issues such as residual neuromuscular blockade or recurarization when the sugammadex dose is administered less than the recommendation [9,10], there have been relatively few studies investigating the impact of overdosing on the efficacy of neuromuscular blockade reversal or the recovery profile from general anesthesia.

Prospective studies comparing overdose and appropriate dosing of sugammadex are scarce, and a few case reports are available, but the results are controversial. One case report described a 7-month-old male undergoing general anesthesia for foreign body removal via bronchoscopy [11]. An accidental overdose of sugammadex at 4.5 mg/kg was administered, resulting in chest wall rigidity, which made ventilation difficult and caused a drop in peripheral oxygen saturation. The situation required reintubation and administration of rocuronium to manage the symptoms effectively.

Another study investigated the pharmacokinetics, safety, and tolerability of single high doses of sugammadex in 13 healthy adults [12]. Except for one case of hypersensitivity reaction, the administration of doses as high as 96 mg/kg was found to be well tolerated.

Based on these available reports, sugammadex appears to have a relatively wide safety margin in adults. This may explain why our study found no significant differences in recovery profiles between administering a fixed dose of 200 mg and a titrated dose. However, studies on the safety of high doses are limited to healthy adult patients, and there have been reports of adverse events in certain patients and pediatric cases. Therefore, the potential risks associated with sugammadex overdose cannot be definitively concluded.

When Sugammadex was approved by the United States Food and Drug Administration (FDA) in 2015, concerns regarding hypersensitivity delayed its approval compared to Europe, and reports of hypersensitivity reactions have since been documented [19,20]. According to some studies, both sugammadex and the sugammadex-rocuronium complex can act as allergens; however, higher levels of free sugammadex molecules in the bloodstream appear to be associated with reactions such as anaphylaxis [21]. Additionally, it has been reported that overdosing sugammadex may increase the incidence of adverse reactions, including anaphylaxis and hypotension [22].

Although our study did not demonstrate significant differences in primary or secondary outcomes between the two groups, considering the widespread availability of quantitative neuromuscular blockade depth monitoring and the potential risks of sugammadex overdosing, it can be speculated that titration of the drug based on patient body weight and comorbidities may offer additional benefits.

This study has several limitations. First, it was conducted at a single center with a relatively small sample size, which may restrict the generalizability of the findings to larger or more diverse populations. Second, when designing this study, we hypothesized that a fixed dose of 200 mg would reduce the time to achieve a normalized TOF ratio of 0.9 by 50%, based on pharmacokinetic data suggesting accelerated recovery with higher doses of sugammadex. However, our results demonstrated a limited difference in total dosage between the two groups, indicating that the assumption of a 50% reduction might have been an overestimation. This highlights the need for further studies to refine these assumptions and explore the relationship between dosing strategies and recovery times more comprehensively. Furthermore, patients with significant comorbidities or those with lower body weight, who may receive relatively higher overdoses, could experience more pronounced effects, highlighting the need for additional studies focusing on these specific subpopulations.

## 5. Conclusions

In conclusion, this study demonstrated that a fixed dose of 200 mg of sugammadex provides comparable efficacy and safety to the recommended weight-based dose of 2 mg/kg for the reversal of moderate neuromuscular blockade in patients undergoing general anesthesia. However, the potential for overdosing, particularly in patients with lower body weight or significant comorbidities, along with the increasing availability of quantitative monitoring tools suggest that a weight-based dosing strategy may offer additional benefits in optimizing patient safety and drug utilization. Further studies with larger sample sizes and diverse populations are needed to validate these findings.

## Figures and Tables

**Table 1:** Baseline Characteristics.

	Group R (n = 10)	Group F (n = 10)	*p*-Value
Age, yr	49.1 ± 13.3	41.4 ± 14.0	0.224
Sex (male/female), n	5/5	6/4	>0.999
Weight, kg	64.2 ± 8.8	68.5 ± 8.8	0.296
Height, cm	164.9 ± 7.7	167.6 ± 6.6	0.406
BMI, kg/m^2^	23.6 ± 3.1	24.3 ± 2.3	0.583
ASA class (I/II), n	2/8	1/9	>0.999
Baseline TOF ratio, %	104.5 (101.8–109.3)	104.5 (102.8–114.8)	0.739
Intraoperative fluids, mL	275.0 (187.5–362.5)	300.0 (200.0–525.0)	0.436
Duration of surgery, min	51.0 ± 44.0	63.4 ± 34.3	0.491
Duration of anesthesia, min	76.5 ± 47.0	88.1 ± 36.7	0.546

Values are expressed as mean ± standard deviation, median (interquartile range), or number of patients. Group R, recommended dose group (2 mg/kg sugammadex), Group F, fixed dose group (200 mg sugammadex); BMI: body mass index; ASA: American Society of Anesthesiologists; TOF: train-of-four.

**Table 2:** Reversal times of TOF ratio and emergence profiles.

	Group R (n = 10)	Group F (n = 10)	*p*-Value
At sugammadex injection			
TOF count	2.0 (1.8–3.0)	1.5 (1.0–2.3)	0.218
BIS value	48.1 ± 6.8	48.4 ± 7.5	0.926
MAC	0.9 (0.7–1.0)	0.9 (0.7–0.9)	0.739
Body temperature, °C	36.2 ± 0.6	36.5 ± 0.5	0.210
Recovery time, min	7.0 ± 1.6	6.1 ± 2.1	0.296
Time from sugammadex injection to			
Normalized TOF ratio ≥ 0.9, min	2.3 (1.8–2.9)	2.0 (1.6–2.6)	0.529
Spontaneous respiration, min	4.8 ± 2.0	4.0 ± 2.3	0.420
Response to verbal command, min	5.8 ± 1.7	5.3 ± 1.9	0.521
Extubation, min	5.9 ± 1.6	5.5 ± 2.0	0.629
Adverse events			
Bradycardia, n	0	0	NA
Airway obstruction, n	0	0	NA
Cough grade (0/1/2/3), n	5/1/3/1	7/1/2/0	0.273

Values are expressed as median (interquartile range), mean ± standard deviation, or number of patients. Group R, recommended dose group (2 mg/kg sugammadex), Group F, fixed dose group (200 mg sugammadex); TOF, train-of-four; BIS: bispectral index; MAC, age-adjusted minimal alveolar concentration; Recovery time, time from the end of surgery to extubation; NA, not applicable.

## Data Availability

The raw data supporting the conclusions of this article will be made available by the authors on request.

## Abbreviations

The following abbreviations are used in this manuscript:

TOF	Train-of-four
ASA	American Society of Anesthesiologists

## References

[1] Dubois P.E., Putz L., Jamart J., Marotta M.-L., Gourdin M., Donnez O. (2014). Deep neuromuscular block improves surgical conditions during laparoscopic hysterectomy: A randomised controlled trial. Eur. J. Anaesthesiol..

[2] Wilcox S.R., Bittner E.A., Elmer J., Seigel T.A., Nguyen N.T.P., Dhillon A., Eikermann M., Schmidt U. (2012). Neuromuscular blocking agent administration for emergent tracheal intubation is associated with decreased prevalence of procedure-related complications. Crit. Care Med..

[3] Keating G.M. (2016). Sugammadex: A review of neuromuscular blockade reversal. Drugs.

[4] Yang L.P., Keam S.J. (2009). Sugammadex: A review of its use in anaesthetic practice. Drugs.

[5] Thilen S.R., Weigel W.A., Todd M.M., Dutton R.P., Lien C.A., Grant S.A., Szokol J.W., Eriksson L.I., Yaster M., Grant M.D. (2023). 2023 American Society of Anesthesiologists practice guidelines for monitoring and antagonism of neuromuscular blockade: A report by the American Society of Anesthesiologists task force on neuromuscular blockade. Anesthesiology.

[6] Fuchs-Buder T., Romero C.S., Lewald H., Lamperti M., Afshari A., Hristovska A.-M., Schmartz D., Hinkelbein J., Longrois D., Popp M. (2023). Peri-operative management of neuromuscular blockade: A guideline from the European Society of Anaesthesiology and Intensive Care. Eur. J. Anaesthesiol..

[7] Batistaki C., Vagdatli K., Tsiotou A., Papaioannou A., Pandazi A., Matsota P. (2019). A multicenter survey on the use of neuromuscular blockade in Greece. Does the real-world clinical practice indicate the necessity of guidelines?. J. Anaesthesiol. Clin. Pharmacol..

[8] Ledowski T., Ong J.S., Flett T. (2015). Neuromuscular monitoring, muscle relaxant use, and reversal at a tertiary teaching hospital 2.5 years after introduction of sugammadex: Changes in opinions and clinical practice. Anesthesiol. Res. Pract..

[9] Fuchs-Buder T., Meistelman C., Alla F., Grandjean A., Wuthrich Y., Donati F. (2010). Antagonism of low degrees of atracurium-induced neuromuscular blockade: Dose-effect relationship for neostigmine. J. Am. Soc. Anesthesiol..

[10] Rodney G., Raju P., Brull S. (2024). Neuromuscular block management: Evidence-based principles and practice. BJA Educ..

[11] Sagun A., Aktas F., Birbicer H. (2017). Chest wall rigidity due to high dose sugammadex. J. Clin. Anesth..

[12] Peeters P.A., van den Heuvel M.W., Heumen E.V., Passier P.C., Smeets J.M., van Iersel T., Zwiers A. (2010). Safety, tolerability and pharmacokinetics of sugammadex using single high doses (up to 96 mg/kg) in healthy adult subjects: A randomized, double-blind, crossover, placebo-controlled, single-centre study. Clin. Drug Investig..

[13] Fuchs-Buder T., Brull S.J., Fagerlund M.J., Renew J.R., Cammu G., Murphy G.S., Warlé M., Vested M., Fülesdi B., Nemes R. (2023). Good clinical research practice (GCRP) in pharmacodynamic studies of neuromuscular blocking agents III: The 2023 Geneva revision. Acta Anaesthesiol. Scand..

[14] Flockton E.A., Mastronardi P., Hunter J.M., Gomar C., Mirakhur R.K., Aguilera L., Giunta F.G., Meistelman C., Prins M.E. (2008). Reversal of rocuronium-induced neuromuscular block with sugammadex is faster than reversal of cisatracurium-induced block with neostigmine. Br. J. Anaesth..

[15] Czarnetzki C., Tassonyi E., Lysakowski C., Elia N., Tramèr M.R. (2014). Efficacy of sugammadex for the reversal of moderate and deep rocuronium-induced neuromuscular block in patients pretreated with intravenous magnesium: A randomized controlled trial. Anesthesiology.

[16] Eleveld D.J., Kuizenga K., Proost J.H., Wierda J.M.K. (2007). A temporary decrease in twitch response during reversal of rocuronium-induced muscle relaxation with a small dose of sugammadex. Anesth. Analg..

[17] Sorgenfrei I.F., Norrild K., Larsen P.B., Stensballe J., Østergaard D., Prins M.E., Viby-Mogensen J. (2006). Reversal of rocuronium-induced neuromuscular block by the selective relaxant binding agent sugammadex: A dose-finding and safety study. J. Am. Soc. Anesthesiol..

[18] Suy K., Morias K., Cammu G., Hans P., van Duijnhoven W.G., Heeringa M., Demeyer I. (2007). Effective reversal of moderate rocuronium-or vecuronium-induced neuromuscular block with sugammadex, a selective relaxant binding agent. J. Am. Soc. Anesthesiol..

[19] Ho G., Clarke R.C., Sadleir P.H., Platt P.R. (2016). The first case report of anaphylaxis caused by the inclusion complex of rocuronium and sugammadex. A&A Pract..

[20] Tsur A., Kalansky A. (2014). Hypersensitivity associated with sugammadex administration: A systematic review. Anaesthesia.

[21] Choi S.C., Han S., Kwak J., Lee J.Y. (2020). Anaphylaxis induced by sugammadex and sugammadex-rocuronium complex-a case report. Korean J. Anesthesiol..

[22] Dunipace D., Sparling J.L., Lee S. (2018). Sugammadex: A New Reversal Drug With the Same Risk-A Continued Need for Neuromuscular Blockade Monitoring. ASA Monit..

